# Harnessing the Phytochemical Diversity of the Genus *Astragalus* for Evolving Cosmeceutical Applications

**DOI:** 10.3390/plants15203236

**Published:** 2026-10-21

**Authors:** Saba Shahrivari-Baviloliaei, Ilkay Erdogan Orhan, Agnieszka Viapiana, Alina Plenis

**Affiliations:** 1 Department of Analytical Chemistry, Faculty of Pharmacy, Medical University of Gdansk, 80-210 Gdansk, Poland; 2 Chemical Analysis Laboratory Lab-Spark Sp. o.o., 81-451 Gdynia, Poland; 3 Department of Pharmacognosy, Faculty of Pharmacy, Lokman Hekim University, 06510 Ankara, Türkiye; ilkayerdoganorhan@gmail.com

**Keywords:** *Astragalus*, antioxidant, cosmetics, compounds, anti-inflammatory, skin

## Abstract

The genus *Astragalus* L. (Fabaceae) comprises approximately 3000 species with a rich history of use in traditional medicine worldwide. While *Astragalus membranaceus* Fisch. ex Bunge is globally recognized as an ingredient in dietary supplements, the cosmetic potential of the broader genus remains largely underexplored. Hence, the current review aims to systematically evaluate 20 *Astragalus* species selected for their documented or prospective applications in the cosmeceutical industry. We examine the diverse phytochemical profiles of these species, highlighting the principal groups of bioactive constituents such as flavonoids (quercetin, kaempferol), saponins (astragaloside IV), and polysaccharides (arabino-3,6-galactan). These compounds have been reported to be associated with critical dermatological benefits, including potent antioxidant activity, skin-lightening effects, anti-inflammatory activity relevant to conditions such as atopic dermatitis, and stimulation of collagen and hyaluronic acid synthesis for anti-aging benefits. By bridging the gap between traditional ethnobotanical use of the selected *Astragalus* species and modern evidence-based scientific formulations, this review provides a roadmap for integrating diverse *Astragalus* species into sustainable, high-performance cosmetic products. Unlike the existing literature, which focuses primarily on *A. membranaceus*, this review provides, to our knowledge, the first systematic comparative analysis of 20 diverse *Astragalus* species, mapping their unique phytochemical markers to specific molecular pathways for advanced cosmeceutical applications.

## 1. Introduction

Medicinal and aromatic plants (MAPs) have been used for various therapeutic purposes since ancient times. Today, as the cosmetic industry expands and consumers increasingly seek natural alternatives, there is growing interest in plant-based formulations that offer greater skin compatibility and potentially fewer adverse effects than synthetic ingredients. Therefore, the cosmetic industry is actively seeking new botanical resources with well-documented dermatological benefits. In this context, plants such as aloe, rose, green tea, and olive have already found widespread use in cosmetics, providing a precedent for investigating other botanically rich genera including *Astragalus*. Olive (*Olea europaea* L.) represents one such example, as its fruits and fixed oil have been used since ancient times. Olive oil is widely incorporated into shampoos, soaps, lotions and creams as a moisturizer and anti-dandruff agent [1]. The plant contains a range of bioactive polyphenolic compounds including catechin, luteolin, rutin, and oleuropein which exhibit strong anti-inflammatory and antioxidant properties that may support skin health [2]. Beyond their therapeutic benefits, many of these plants also contribute to the sensory properties and consumer appeal of cosmetic formulations. The scent of roses (*Rosa* L.) has been appreciated since ancient times and continues to be incorporated into perfumes, creams, and lotions for its aromatic as well as anti-inflammatory and antiseptic properties [3]. Few plants, however, have a longer or more well-documented history in skincare than *Aloe vera* (L.) Burm. f., which has been used by humans for thousands of years. Although *A. vera* gel may rarely cause skin allergies or eczema, it is widely used in the cosmetic and health industries, with applications in moisturizers, creams, cleansers, lotions, shampoos, toothpastes, and deodorants, and the FDA has approved it as a cosmetic ingredient [4]. Black and green tea (*Camellia sinensis* L.), which contain catechins, epigallocatechin gallate, chlorogenic acid, and other polyphenols, while consumed daily, has several cosmetic-related effects such as antioxidant, antibacterial, anti-inflammatory, and hair-strengthening properties [5].

The genus *Astragalus* L. (Fabaceae) comprises approximately 3000 species distributed across nearly all parts of the world [6]. Many species of this genus have been reported to exhibit noteworthy biological activities of relevance to human health, including antidiabetic, antimicrobial, anticancer, analgesic, antifatigue, anti-infective, anti-inflammatory, antiperspirant, antianorexic, antioxidant and tonic activities [7,8,9,10]. Extracts of some *Astragalus* species, particularly *A. membranaceus* in the first place, can also be found in dietary supplements [11,12]. *A*. *membranaceus* is used to support immune function, manage immune-related diseases, and alleviate the adverse effects of chemotherapy [13]. In one study, *Astragalus glycyphyllos* L. Was reported to exhibit marked anti-tumor activity [14]. Most species within this genus remain understudied and warrant further preclinical and clinical investigation.

Among the diverse biological activities of medicinal and aromatic plants relevant to cosmetic applications, their antioxidant, anti-inflammatory, antibacterial, moisturizing, and wound-healing activities, as well as tyrosinase (TYR) inhibition, hold particular significance [15]. TYR is a key enzyme involved in melanogenesis and enzymatic browning, making its inhibitors valuable ingredients in depigmenting and skin brightening/lightening products [16]. Interest in the therapeutic potential of *Astragalus* species for skin disorders and cosmetic applications has grown in recent years. So far, approximately 300 compounds have been isolated from various *Astragalus* species, including formononetin, calycosin, and ononin, which have been associated with a range of pharmacological activities, such as anticancer, anti-inflammatory, and antioxidant activities [17]. Most existing reviews on *Astragalus* focus mainly on *A. membranaceus* and its systemic pharmacological effects, including its reported adaptogenic and immunomodulatory properties. Despite this chemical richness, most *Astragalus* species remain understudied. Thus, the present review systematically evaluates the phytochemical diversity and dermatological properties of 20 species in this genus, to our knowledge, for the first time. Our goal is to identify promising candidates for natural cosmetic formulations by correlating specific secondary metabolites with clinical and preclinical evidence of skin benefits including the anti-melanogenic, anti-inflammatory, and anti-aging effects of the selected *Astragalus* species. Literature was searched using Google, Google Scholar, Web of Science, Scopus, and PubMed for the period 1990–2026 using keywords such as “*Astragalus* compounds,” “flavonoids in cosmetics,” “cosmetic products with *Astragalus*,” and “*Astragalus* spp. + cosmetic.”

## 2. Chemical Composition of *Astragalus* Species

The various biological effects of medicinal plants can be attributed to their diverse chemical constituents. To date, the chemical compositions of species such as *A. membranaceus*, *A. cicer*, *A. glycyphyllos*, *A. strictispinus*, and *A. macrocephalus* subsp. *finitimus* have been studied, and about 300 compounds have been isolated from different *Astragalus* species including polysaccharides and lignin [18], flavonoids, alkaloids and coumarins [19], saponins, steroids, organic acids, essential oils [20], and other bioactive compounds [21]. Flavonoids are among the bioactive compounds of interest in cosmetic formulations. They are used in skincare products due to their soothing and antioxidant properties [22]. Among them quercetin, cyanidin-3-glucoside, and rutin are widely used in the cosmetics industry because they protect the skin from ultraviolet (UV) light [23,24]. Moreover, flavonoids are among the main secondary metabolites of this genus [25,26]. Several flavonoids, including alfalone, cajanin, calycosin, quercetin, kaempferol, sulfuretin, ampelopsin, and 7-hydroxy-3′,5′-dimethoxyisoflavone, have been identified in this genus, with quercetin and kaempferol reported as major constituents. A series of flavonoids have also been identified for the first time in species of *Astragalus*, such as complanatin, falcosides C and D, astraciceran, and astragaluquinone, some of which have names derived from the genus [27,28,29,30,31,32].

As major bioactive constituents of *Astragalus* spp., saponins are extensively utilized in cosmetic formulations due to their inherent surfactant, foaming, and emulsifying properties [33]. Among them, azukisaponin V, cloversaponin III, astrachrysoside A, astraoleanosides H, cycloastragenol, cycloascauloside B, brachyoside B, and astrasieversianin X have been identified in various species of *Astragalus* [12,34,35,36,37,38,39,40]. However, the major saponins include astragalosides, which are key active compounds in *A. membranaceus* and have been reported to protect human skin fibroblasts against UVA-induced photoaging [41].

Polysaccharides represent another important group of bioactive constituents found in *Astragalus* species, with potential applications in cosmetic formulations [42]. Although polysaccharides are widely distributed primary metabolites of many plants, numerous studies on *Astragalus* polysaccharides (APS) have revealed their therapeutic properties such as inhibiting tumor growth and viral replication, antimutations, anti-inflammation, immunomodulatory, cardioprotective, neuroprotective, and antidiabetic properties [43,44]. APSs (e.g., heteropolysaccharides, glucans, neutral polysaccharides, and acidic polysaccharides) offer practical benefits in cosmetic formulations as thickeners, emollients, emulsifiers as well as gelling agents [45,46]. Some APSs possess moisturizing and hydrating, anti-aging, anti-inflammatory, and anti-collagenase properties or act as anti-spot and skin-tightening agents by inhibiting TYR [47,48]. Arabino-3,6-galactan, a type II arabinogalactan identified in *Astragalus* [49], exemplifies this dual utility. Already used in industry as a moisturizing and gelling agent [50], it highlights the potential of *Astragalus* polysaccharides as multifunctional ingredients capable of both structuring a product and delivering active skin benefits.

## 3. *Astragalus* Species

### 3.1. A. membranaceus Fisch. ex Bunge

One of the most well-known species in this genus is *A. membranaceus* Fisch. ex Bunge. Its roots hold a prominent place in traditional Chinese medicine, where they are regarded as one of the most important adaptogenic herbal medicines and are used to treat chronic diarrhea, fatigue, and abnormal uterine bleeding [11]. This species has been studied more extensively than other *Astragalus* species, owing to its widespread use as an immune booster and dietary supplement worldwide. The plant is rich in flavonoids, saponins, polysaccharides as well as trace elements and amino acids. Calycosin-7-glucoside, calycosin, and formononetin are the major isoflavonoid constituents of this plant [51,52]. Astragaloside IV, a triterpene glycoside with potent anti-inflammatory activity and protective effects on the cardiovascular system, kidneys, lungs, and brain, as well as in gynaecological diseases, is also one of the main components of *Astragalus* species. This potent anti-inflammatory effect is associated with the modulation of intracellular signaling, including the inhibition of nuclear factor kappa B (NF-κB) activation and reduction in the expression of pro-inflammatory cytokines such as tumour necrosis factor-alpha (TNF-α) and interleukin-6 (IL-6), suggesting a mechanism involved in reducing skin inflammation. Other pharmacological effects are associated with numerous signalling pathways, including the Nrf2 antioxidant signalling pathway [53,54]. APS, e.g., APS-I and APS-II, have been identified in the roots and have exhibited anti-tumour properties [55,56]. Formononetin is a natural isoflavonoid with anti-tumor properties that can be extracted from *A. membranaceus* [57]. It also has an estrogenic effect, which may play a role in keeping the skin healthy by maintaining moisture and reducing wrinkles, in addition to its antioxidant properties [58]. In an ex vivo study using mice, astragaloside IV was reported to promote hair growth, strengthen hair follicles, and reduce hair loss. It also increased the levels of collagen and fibronectin in the extracellular matrix of the cells. This molecule significantly improved wound closure and re-epithelialization in mice with streptozotocin-induced diabetic ulcers compared with control mice [59]. The anti-inflammatory effects of *A. membranaceus* have been demonstrated in several studies, including the alleviation of atopic dermatitis symptoms through the reduction of inflammatory mediators and suppression of the NF-κB and MAPK pathways [60]. Choi and Kim [61] demonstrated that astragaloside-IV suppresses inflammatory responses by inhibiting the phosphorylation of NF-κB (p65) and IκBα, as well as that of MAPK components (ERK, JNK, and p38), in a DSS-induced colitis model. This was accompanied by reduced expression levels of pro-inflammatory cytokines (TNF-α, IL-1β) and oxidative stress markers (iNOS, COX-2, ROS). Additionally, Chang et al. reported that a saponin blend from *A. membranaceus* increased collagen synthesis and reduced UVB-induced MMP-1 expression in human skin cells [62]. In addition, in vivo and clinical studies have shown that saponins from *A. membranaceus* stimulate the synthesis of collagen and hyaluronic acid. They also increase skin elasticity and reduce wrinkles and melanin levels, thereby potentially contributing to skin rejuvenation [62]. Furthermore, studies have demonstrated the anti-melanogenic efficacy of calycosin and its ability to suppress pigmentation and TYR activity. Taken together, these data suggest that calycosin is a promising candidate for development as a skin-lightening cosmetic ingredient. Studies of calycosin report reduced expression of microphthalmia-associated transcription factor (MITF), and modulation of the protein kinase A (PKA)/cAMP response element-binding protein (CREB) and mitogen-activated protein kinase (p38 MAPK) signaling pathways; these constituent-level findings should not be assumed to establish the same mechanism for the whole plant extract [63]. Hence, this plant may have potential applications in cosmetic products such as creams and shampoos and is reported to be incorporated into various commercially available formulations, including creams, masks, and toners.

### 3.2. A. sinicus L.

*A. sinicus* plays an important role in agriculture, where it is grown as a winter cover crop or green manure in paddy fields [64]. Flavonoid glycosides (such as myricetin, among others) and flavonols (such as ampelopsin, among others) with marked antioxidant potential have been identified in the seeds of this plant [30]. However, results from in vitro and in vivo studies indicate that acetone extracts of *A. sinicus* seeds displayed markedly higher antioxidant activity than the aqueous extracts, with approximately fourfold higher total phenolic content and threefold higher flavonoid content [65]. Choi et al. [66] experimentally demonstrated that the acetone extract of *A. sinicus* seeds directly inhibits key skin-degrading enzymes in a concentration-dependent manner. Specifically, TYR activity was inhibited by up to 93.8% at 20 mg/mL, elastase by up to 97.5% at 200 μg/mL, and collagenase by up to 99.0% at 300 μg/mL. These in vitro enzymatic inhibitory effects were further corroborated by an 8-week clinical trial, in which a cream containing 0.2% of the extract significantly improved pigmentation (28.56 MI), coarseness (18.45 R-value), elasticity (64.5 Ur/Uf), and moisture (55.2 AU) indices compared with baseline [66]. Thus, the phenolic-rich composition of this extract (e.g., myricetin, and ampelopsin) provides a plausible basis for its antioxidant activity. The cosmetic potential of *A. sinicus* seed extract appears to be primarily associated with direct inhibition of TYR, elastase, and collagenase, suggesting potential effects on melanogenesis and extracellular matrix degradation pathways, in addition to phenolic-mediated antioxidant activity. By inhibiting elastase and collagenase, the extract may thereby help limit extracellular matrix degradation and potentially mitigate processes associated with photoaging. Consistent with this enzyme-inhibitory activity, a recent network pharmacology study predicted that bioactive compounds from *A. sinicus* may target the MAPK signaling pathway [67]. Nevertheless, because this in silico prediction has not been experimentally validated for the whole extract, the MAPK/AP-1 axis should be considered a reasonable hypothesis rather than a confirmed mechanism. Direct experimental evidence (e.g., via Western blot analysis or pathway-specific inhibitors) is therefore needed to substantiate this link in future investigations.

In addition, in an in vitro study conducted by Jeon et al. [68], the potential of *A. sinicus* to reduce damage to human hair follicle dermal papilla cells (HFDPCs) induced by dihydrotestosterone (DHT) was evaluated. The extract was found to promote wound healing, increase alkaline phosphatase expression in DHT-damaged HFDPCs, and restore ATP levels by recovering mitochondrial membrane potential in damaged cells. Moreover, this plant significantly increased the phosphorylation levels of AKT (also known as protein kinase B, PKB) and extracellular signal-regulated kinase (ERK) thereby suggesting activation of the AKT/ERK pathway; it also modulated the Wnt signaling pathway in HFDPCs [68]. Taken together, these findings suggest that *A. sinicus* may have potential as a source of bioactive compounds for hair-loss-related cosmetic applications. Therefore, it could serve as a natural source of bioactive ingredients for the cosmetic industry.

### 3.3. A. exscapus Subsp. Transsilvanicus (Janka) Nyar.

*A. exscapus* subsp. *transsilvanicus* is a rare and endangered plant endemic to the Transylvanian Plateau in Romania [69]. Its primary bioactive phytochemicals include polysaccharides, lipids, and phenolic compounds, with astragaloside IV identified as a predominant bioactive marker [70,71]. Moreover, in the study conducted by Szabo et al. [71], linoleic, palmitic, oleic, and α-linolenic acids were found at the highest concentrations among the fatty acids analyzed. In a previous in vitro study on *A. exscapus* subsp. *transsilvanicus* [71], its antioxidant, antimicrobial, and cytotoxic activities were evaluated and the extracts exhibited significant antimicrobial activity against *Staphylococcus aureus* and *Staphylococcus epidermidis*. Moreover, cell proliferation assays revealed a significant inhibitory effect on the proliferation of B16F10 melanoma cells [71]. Melanoma is a highly aggressive form of skin cancer that can produce significant amounts of melanin through a process called melanogenesis, and melanin pigments can reduce the effectiveness of immunotherapy and radiotherapy [72]. Therefore, due to ROS scavenging and antimicrobial activities, along with its potential interference with melanin production pathways, the plant extract could potentially be incorporated into cosmetic formulations such as creams, cleansers or soaps.

### 3.4. A. caraganae Fisch. & C.A.Mey.

*A. caraganae* is another species that has been reported to possess anti-TYR and antioxidant activities, and contain compounds such as rutin, chlorogenic acid, isoquercitrin, and delphinidin-3,5-diglucoside [73]. As flavonoid derivatives, rutin and delphinidin-3,5-diglucoside offer diverse biological benefits, including antioxidant and anticancer activities. Moreover, chlorogenic acid is a polyphenol with well-documented antioxidant and anti-inflammatory properties. As a glycosylated derivative of the flavonol quercetin, isoquercitrin has been reported to retain the antioxidant and anticancer properties of its parent compound while also showing promise for the management of allergic conditions [74,75,76]. Thus, this species may warrant further investigation for potential applications in anti-blemish or photoprotective formulations. Extracts from this plant have been shown to exert cytostatic effects on human dermal cells at high concentrations [74]. The scientific literature directly evaluating *A. caraganae* with respect to NF-κB pathway modulation and cytokine reduction is extremely limited compared with the evidence available for major well-studied species such as *A. membranaceus* or its active constituents, such as astragaloside IV and APSs. Although direct evaluation of inflammatory mediators such as TNF-α or IL-6 has not been reported for *A. caraganae*, its constituent phenolic compounds, particularly chlorogenic acid and rutin, have been extensively studied in other experimental models. Chlorogenic acid has been shown to suppress LPS-induced NF-κB activation and JNK/AP-1 signaling, thereby reducing COX-2 expression and PGE_2_ release in macrophages [40,77]. Additionally, chlorogenic acid inhibits p38 MAPK phosphorylation in LPS-stimulated RAW264.7 cells [78]. Similarly, rutin has been reported to downregulate the NF-κB and MAPK (ERK/JNK/p38) pathways, leading to reduced expression of pro-inflammatory cytokines including TNF-α, IL-6, and IL-1β, as well as iNOS and COX-2, in various inflammatory and cancer cell models [79]. Collectively, these findings suggest that *A. caraganae* may have anti-inflammatory and photoprotective potential, potentially mediated in part by the combined activity of these well-characterized phenolic compounds, although direct mechanistic validation using the whole extract remains to be performed. Therefore, the cosmetic potential of this species may extend beyond depigmenting effects to include antioxidant-mediated photoprotective and anti-inflammatory properties. However, mechanistic studies investigating intracellular signaling pathways in *A. caraganae* remain limited and warrant further exploration. Therefore, the cosmetic potential of this species may extend beyond depigmenting effects to include antioxidant-mediated photoprotective and anti-inflammatory properties. However, the intracellular signaling pathways involved remain insufficiently characterized and warrant further investigation.

### 3.5. A. verus Olivier

The essential oil of this plant contains compounds such as thymol, palmitic acid, and phytol [80]. Thymol displays diverse biological activities—antifungal, antioxidant, antibacterial, anti-inflammatory, wound-healing, analgesic, acaricidal, and radioprotective effects. Its anti-inflammatory mechanism involves suppression of NF-κB and MAPK signaling in lipopolysaccharide (LPS)-stimulated mECCs, with consequent downregulation of TNF-α, IL-1β, and IL-6. It is used as a flavouring agent, and as an ingredient in toothpastes, mouthwashes, soaps, shampoos, topical ointments, and deodorants [81,82,83]. Phytol, a diterpene alcohol identified among the constituents of *A. verus*, is used in cosmetics because of its antioxidant and anti-aging properties [84]. A previous in vitro study investigating the potential dermatological applications of *Astragalus* species reported antimicrobial activity of *A. verus* and its potential for topical application against infection with *Trichomonas vaginalis*, the most common non-viral sexually transmitted infection which can also cause complications during pregnancy [85]. In addition to its antimicrobial and immunomodulatory properties, this species has also been reported to promote hair growth and relieve symptoms of skin and nail infections (dermatophytosis). Thus, it may have potential applications in products such as shampoos and cleansers. It should be noted that, according to the results of clinical trials conducted in 1987, the use of oleic, lauric, palmitic, myristic, and stearic acids in cosmetics was considered safe [86].

### 3.6. A. gummifer Labill.

This species grows in Iran, Türkiye, and Syria. The gum of *A. gummifer* is one of the most widely used natural gums worldwide, and possesses softening and hydrating properties, while it has also been reported as to have analgesic properties in traditional Iranian medicine. In addition to its analgesic properties, the gum is used as a general tonic and laxative, and to treat coughs and chapped lips in Iranian folk medicine [87]. In Jordanian folk medicine, it is also widely employed to treat stomachaches and coughs [88]. This species has been reported to exhibit anti-inflammatory and immunomodulatory effects [89]. Moreover, this plant has been reported to accelerate wound healing [90]. Its wound-healing ability may be related to its structural similarity to chitosan, another arabinogalactan-containing biopolymer and promoting re-epithelialization, angiogenesis through increased vascular endothelial growth factor (VEGF) expression, collagen and fibronectin deposition, and polarization of macrophages towards the M2 repair phenotype, as these pathways collectively accelerate skin healing and matrix remodeling in vivo [60]. With its softening, hydrating, and thickening properties, the gum of *A. gummifer* is used in creams and lotions, and as a viscosity-enhancing agent in cosmetic and pharmaceutical preparations [91].

### 3.7. A. plumosus Willd. Var. Krugianus Freyn & Bornm.

*A. plumosus* var. *krugianus* is a species endemic to Türkiye that contains cycloartane glycosides such as askrugianoside A and oleifolioside B. These types of glycosides are known to display strong protective effects against oxidative stress, which may slow the aging process. Accordingly, several previous studies indicated that some of the cycloartane glycosides in this plant have the potential to protect human skin fibroblasts from oxidative stress [92,93]. The protective effects of such triterpene glycosides have been associated with enhancement of cellular antioxidant defense systems. These compounds may exert their protective effects through activation of cellular antioxidant defense systems, including nuclear factor erythroid 2-related factor 2 (Nrf2)/antioxidant response element (ARE) signaling and upregulation of phase II antioxidant enzymes, thereby potentially reducing ROS levels and helping to preserve extracellular matrix integrity [94]. Therefore, this species warrants further investigation for potential skin rejuvenation applications.

### 3.8. A. glycyphyllos L.

In some countries and regions, such as Bulgaria, France, Ukraine, and the Caucasus region, *A. glycyphyllos* is used to manage sexually transmitted infections, fungal skin infections, and dermatitis, and is valued for its emollient, refreshing, and anti-inflammatory properties [9,39]. Extracts from different parts of the plant, especially its leaves, have exhibited marked antioxidant, antibacterial, and antifungal properties that may be beneficial in cosmetic and healthcare products [40,95]. The metabolite profile of *A. glycyphyllos*, which includes quercetin, rutin, vitamins C and E, zinc, iron, and fatty acids (stearic, palmitic, and linoleic acids), contains several compounds of potential relevance to cosmetic applications. These compounds may contribute to various functions in cosmetic formulations, including anti-aging, thickeners, emulsifiers, softeners, and moisturizers, while also potentially exerting anti-inflammatory and skin-soothing effects relevant to conditions such as eczema and psoriasis [40]. The skin benefits of *A. glycyphyllos* may be associated with its rich content of flavonoids and other antioxidant compounds, including quercetin and rutin, which have been reported to modulate key cell signaling pathways. These compounds have been reported to NF-κB and MAPK/AP-1 activation, thereby reducing the production of pro-inflammatory cytokines and MMP-1 expression, while enhancing antioxidant defenses through activation of the Nrf2 pathway. In addition, the presence of vitamin C may support collagen biosynthesis, and essential fatty acids may contributed to the maintenance and repair of the epidermal barrier. Collectively, these mechanisms may provide a mechanistic rationale for its traditional use in inflammatory and fungal skin disorders [96,97,98].

### 3.9. A. mongholicus Bunge

*A. mongholicus* is a perennial herb used in traditional Chinese medicine for various therapeutic purposes, including strengthening the immune system and heart, as well as for its anticancer, antidiabetic, and antiviral properties [99]. Botanically similar to *A. membranaceus* and sometimes classified as its synonym, *A. mongholicus* is recognized for its immunomodulatory, antioxidant, and anti-inflammatory properties. Its saponin and flavonoid constituents may contribute to its relevance for anti-aging, anti-inflammatory, and anti-acne cosmetic applications [100].

Fibrosis is a pathological tissue repair response that can also occur in the skin. This process is associated with several factors such as oxidative stress, inflammation, fibroblast proliferation, and excessive extracellular matrix deposition [101]. A previous study demonstrated the antifibrosis effects of this plant. Another study showed that it reduces skin inflammation as well as the severity of psoriasis and scaling [60]. Based on these results, this species may be considered for further investigation for potential use in cosmetic formulations. This plant may exert its dermal and antifibrotic effects through the modulation of key inflammatory and fibrogenic signaling pathways. Furthermore, its antifibrotic activity has been associated with suppression of the transforming growth factor-beta (TGF-β)/Smad signaling cascade, leading to decreased fibroblast activation and collagen overproduction. Activation of the Nrf2 pathway may further enhance antioxidant defenses and limit oxidative stress-induced skin damage; however, this mechanism requires direct validation for the preparation under discussion. These mechanisms suggest potential application of this species in anti-aging, anti-inflammatory, and antifibrotic cosmetic formulations [102,103].

### 3.10. A. monspessulanus L.

*A*. *monspessulanus* comprises three subspecies and is widely distributed in semiarid and arid regions, as well as in the Tell region of the Algerian Atlas [104,105]. Benslama et al. [106], through in vitro and in silico investigations, demonstrated the antioxidant, photoprotective, and anti-TYR potential of *A. monspessulanus* extract. Phytochemical analysis revealed the presence of flavoalkaloids and flavonoids [105,106]. These constituents are associated with anti-inflammatory, anti-TYR, photoprotective, and antioxidant activities [107,108,109]. For instance, cosmosiin exhibits antiviral and antioxidant properties [106,107], while trifolin demonstrates anticancer and antifungal effects [108]. Moreover, rutin, a well-characterized flavonoid, has been reported to protect against ultraviolet radiation and oxidative stress in skin models [109,110]. It has been shown to increase the sun protection factor (SPF) of sunscreen formulations, positioning it as a safe and effective ingredient in cosmetic products such as sunscreens [111]. With its combination of photoprotective, antioxidant, and anti-inflammatory flavonoids, *A. monspessulanus* may warrant further investigation for the development of cosmetic formulations.

### 3.11. A. cruciatus Link.

*A. cruciatus* (synonyms: *A. radiatus*, *A. aristidis*, *A. corrugatus*, and *A. trabutianus*) is a notable source of bioactive compounds. Recently, Tedjani et al. [112] demonstrated the antihyperglycemic potential of galactomannan-rich seeds of *A. cruciatus* through the inhibition of α-amylase and α-glucosidase. Furthermore, the species contains several phytoconstituents, including nicotiflorin, daucosterol, and narcissin [113], which contribute to its documented in vitro antioxidant and anti-inflammatory properties [114,115]. The observed bioactivities may involve the coordinated regulation of cellular signaling networks. The glycosides may activate Nrf2 signaling, thereby facilitating ARE-dependent upregulation of cytoprotective enzymes (e.g., HO-1 and NQO1) and thereby potentially reducing oxidative stress. Additionally, the phenolic constituents may exert anti-inflammatory effects through suppression of NF-κB pathway activation—specifically, by preventing IκBα degradation and p65 nuclear translocation—thereby potentially reducing the synthesis of pro-inflammatory cytokines [116,117]. In addition, polysaccharide fractions, including galactomannan from the seed mucilage, may contribute to antioxidant and anti-inflammatory activities by modulating the NF-κB and Nrf2 pathways [118].

### 3.12. A. dasyanthus Pall.

This species (commonly known as the woolly-flowered milkvetch) is a perennial medicinal plant included in the European Red Book and widely cultivated for medicinal purposes. Pharmacologically, the species is recognized for its sedative, hypotensive, and antioxidant activities. Its phytochemical profile is characterized by a diverse array of constituents, including flavonoids, organic acids, carbohydrates, and triterpene saponins such as glycyrrhizin [119,120]. These compounds, particularly organic acids, are extensively utilized in the cosmeceutical industry for their anti-aging and pH-stabilizing properties. For instance, tartaric acid serves as an effective anti-inflammatory and antioxidant agent in various dermatological and medicinal formulations [121]. Relevantly, flavonoids such as kaempferol and quercetin, which activate the Nrf2/HO-1 pathway and suppress NF-κB-mediated inflammation [122], are highly valued in the cosmetics industry. Given these properties, they are frequently incorporated into creams, serums, moisturizers, and other personal care products [121]. Kaempferol reduces the production of free radicals and apoptosis and increases the expression of the protein HO-1 and melanin synthesis in PIG1 cells. This flavonoid also plays a role in reducing inflammation [123]. The potential of this species in cosmetics is supported by the functional properties of its key compounds. For instance, malic acid acts as a skin pH regulator in cosmetic formulations and is used at low concentrations in a wide range of products [124]. Similarly, citric acid offers both antioxidant and anti-inflammatory effects; a study by Bernstein et al. [125] demonstrated that it increases the thickness of the viable epidermis and the content of dermal glycosaminoglycans [125]. Furthermore, research has identified specific compounds with targeted benefits, such as calycosin, which was found to be a potent TYR inhibitor [126,127]. Given these diverse bioactive compounds and their established applications, this species represents a promising option for use in cosmetic products.

### 3.13. A. melanophrurius Boiss.

Endemic to Türkiye, *A. melanophrurius* contains a range of compounds widely used in cosmetics—including ferulic acid, rosmarinic acid, chlorogenic acid, *p*-coumaric acid, and verbascoside (syn. acteoside), which contribute to its potent antioxidant and anti-TYR effects [128,129]. Ferulic acid has antioxidant, anticancer, anti-aging, anti-inflammatory, and antibacterial properties and is widely used in the food, pharmaceutical, and cosmetic industries, particularly in skincare products [130]. The antioxidant and anti-inflammatory effects of these phenolics are mainly mediated through activation of the Nrf2/HO-1 pathway and suppression of NF-κB signaling [131,132]. TRPV3, an ion channel expressed in the skin, is upregulated during UVB-induced inflammation, and NF-κB signaling is also activated by UV radiation, leading to the release of pro-inflammatory factors and increased proliferation of melanocytes and keratinocytes in the skin. Rosmarinic acid, as a widely known phenolic acid, plays a role in skin health and youth by reducing and controlling the signaling of this pathway and selectively inhibiting TRPV3. Qu et al. [133] demonstrated that rosmarinic acid directly and selectively inhibits the TRPV3 channel by binding to residues T636 and T665, leading to a reduction in single-channel open probability without altering unitary conductance. This inhibition subsequently suppresses the downstream NF-κB signaling pathway, attenuating the expression of p-P65, TNF-α, and IL-6, and thereby alleviating skin inflammation [133]. In addition, several of these phenolics directly inhibit TYR activity and reduce MITF-mediated melanogenesis, supporting their potential use as skin-lightening and anti-photoaging agents in cosmetic formulations [133]. *p*-Coumaric acid exhibits antioxidant activity by scavenging ROS and activating Nrf2-mediated antioxidant responses, exerts anti-inflammatory effects by reducing TNF-α levels and modulating NF-κB signaling, and demonstrates strong anti-TYR activity, particularly against human TYR, with an IC_50_ of 3 μM—although the relative potency of *p*-coumaric acid, arbutin, and kojic acid depends on the assay conditions and should therefore be interpreted cautiously [134]. Relevantly, Verbascoside, a phenylpropanoid glycoside found in various plant species including *Astragalus*, is valued in the pharmaceutical and cosmetic industries for its antioxidant, anti-inflammatory, and photoprotective properties. Its stability in topical formulations and potential for wound healing have also been documented [135]. Hence, this species has the potential to be used in creams, lotions and other cosmetic products.

### 3.14. A. gombiformis Pomel

*A. gombiformis* is a plant endemic to Algeria, where it is traditionally used to treat scorpion or snake bites. Previous studies have shown that it possesses antioxidant and antibacterial activities, exhibits anti-inflammatory effects comparable to those of diclofenac and ketoprofen, and demonstrates a high UV absorption capacity. These bioactivities have been associated with the plant’s phytochemical profile, including cirsiliol and silymarin. Cirsiliol exerts anti-inflammatory effects by suppressing the activation of the NF-κB and MAPK signaling pathways, as evidenced by reduced phosphorylation of IκBα, p65, IKKα/β, p38, ERK, and JNK, which in turn decreases the production of pro-inflammatory cytokines such as TNF-α, IL-6, and IL-1β, as well as inflammatory mediators including iNOS and MCP-1 [136,137,138]. Additionally, cirsiliol acts as a selective inhibitor of 5-lipoxygenase, further contributing to its anti-inflammatory profile. Silymarin, a standardized flavonolignan complex derived from *Silybum marianum*, is widely used in skincare and cosmetic products due to its potent antioxidant effects and ability to reduce UVB- and chemically-induced damage that may contribute to skin cancer. Mechanistically, silymarin activates the AHR-Nrf2 pathway, upregulating endogenous antioxidant defenses and reducing UV- and pollution-induced ROS generation. Furthermore, silymarin inhibits the activity of collagenase and elastase, while its activation of cannabinoid receptor type 2 (CB2R) on mast cells triggers the release of β-endorphin, providing an additional anti-inflammatory and analgesic mechanism [139]. Collectively, these findings suggest that cirsiliol and silymarin as key bioactive contributors to the dermatological and photoprotective potential of *A. gombiformis*. Given its UV-absorbing and anti-inflammatory properties, this species can be considered a promising candidate for cosmetic formulations, especially photoprotective formulations such as sunscreens.

### 3.15. A. macrocephalus Subsp. Finitimus (Bunge) D.F.Chamb.

The methanol extract of *A. macrocephalus* subsp. *finitimus* demonstrated notable antioxidant and tyrosinase-inhibitory activities. The leaf extract, which contained the highest levels of phenolic compounds (37.68 mg GAE/g) and flavonoids (39.23 mg QE/g), exhibited the strongest effects across all assays, including DPPH (IC_50_ = 2.65 mg/mL), ABTS (IC_50_ = 1.45 mg/mL), and TYR inhibition (IC_50_ = 1.02 mg/mL). These activities are potentially associated with the presence of hyperoside (1828.94 μg/g), apigenin (181.90 μg/g), and *p*-coumaric acid (33.12 μg/g) as the dominant phenolic constituents [140]. Some constituents reported in this species have been shown antioxidant or anti-inflammatory activity in other models, including effects involving Nrf2/HO-1 or NF-κB signaling; these pathways have not been established as mechanics of the whole *A. macrocephalus* subsp. *finitimus* extract. Among the bioactive constituents, hyperoside is reported to possess antioxidant, anti-inflammatory, and anti-wrinkle properties, making it of potential relevance in cosmetic and skincare products, particularly anti-wrinkle creams [141]. Apigenin, another bioactive constituent of *A. macrocephalus*, exhibits a broad spectrum of activities including antioxidant, anticancer, anti-inflammatory, anti-tyrosinase, antibacterial, and antiviral effects [142] and, together with p-coumaric acid, has been reported to suppress melanogenesis through the downregulation of TYR and MITF expression [143,144]. It is used in many cosmetic and health products, including sunscreens, anti-wrinkle creams, and mouthwashes [14q2]. In summary, the findings on *A. macrocephalus* subsp. *finitimus* suggest that this species may represent a potential source of natural bioactive compounds with relevance to both pharmaceutical and cosmeceutical applications. However, further studies are needed to confirm its cosmetic efficacy, elucidate the underlying mechanisms, and establish its topical safety.

### 3.16. A. siliquosus Boiss.

Zarei and Babaei [145] reported that the methanol extract of *A. siliquosus* exhibits significant TYR inhibitory and antioxidant activities, with the flower extract showing the most potent effects (97% TYR inhibition, IC_50_ = 1.58 mg/mL; DPPH EC_50_ = 0.089 mg/mL). The mechanism of TYR inhibition was characterized as mixed-type inhibition with both uncompetitive and noncompetitive characteristics [146]. While direct enzyme inhibition contributes to anti-melanogenic effects, the regulation of melanogenesis may also involve suppression of microphthalmia-associated transcription factor (MITF), a key regulator of tyrosinase expression [147], along with activation of the Nrf2/HO-1 antioxidant pathway to mitigate oxidative stress [147]. Collectively, these findings suggest that *A. siliquosus* is as a promising candidate for skin-lightening applications, although future studies should focus on isolating and characterizing the specific bioactive constituents responsible for the activities of the most active plant parts (flowers and stems) for cosmetic development.

### 3.17. A. gyzensis Bunge

*A. gyzensis* is a small annual herb native to the Sahara, characterized by creeping cylindrical stems covered with whitish hairs and leaves bearing seven broad green leaflets. Locally known as “dilia”, it is traditionally used in Algerian folk medicine for its reported analgesic, antiseptic, and anti-inflammatory properties and to treat snakebites, scorpion stings, diabetes, infertility, digestive and respiratory disorders, cancer, gum inflammation, back pain, and jaundice, as well as being used as a galactagogue and uterine tonic [148]. The seeds contain mucilage, phenolic compounds, proteins, and uronic acid [148]. Some of these constituents, such as mucilage and uronic acid are known to function as moisturizers by forming a hydrophilic layer that may help reduces transepidermal water loss [146]. Given these properties, *A. gyzensis* have potential applications in cosmetic applications. However, further research is required to validate its efficacy in cosmetic formulations.

### 3.18. A. strictispinus Boiss.

The literature on *A. strictispinus* chemical composition and its biological activity is limited. This plant is native to Turkey. Aydogan et al. [70] investigated ethanolic extracts of roots and found flavonoids, lignans, and steroidal glycosides as the main constituent classes. Among the identified compounds, cycloasalgenin has been reported to exhibit anti-inflammatory properties, while cycloastragenol activates telomerase, indicating its potential relevance to anti-aging potential [7,149,150]. Hesperidin has been reported to provide UV protection [151,152], and salicylic acid has keratolytic, comedolytic, and anti-inflammatory properties [153,154]. Moreover, ethanolic extracts of *A. strictispinus* roots showed anti-inflammatory activity [70]. Another study reported that ethanolic extracts of *A. strictispinus* leaves showed their antibacterial activity against *Staphylococcus aureus* [12]. Collectively, these findings suggest that *A. strictispinus* may represent a potential source of bioactive compounds of interest to the cosmeceutical industry. However, more studies focusing on this plant are needed to better understand its chemical profile and biological activity.

### 3.19. A. gossypinus Fisch.

*A. gossypinus*, native to Iran, Türkiye, Lebanon, and Iraq, grows mainly in the temperate biome. It has been used in Iranian traditional medicine and cosmetics due to its pharmacological and biological activities [155]. This species is a major source of high-quality tragacanth gum (locally known as katira in Persian), which is a highly complex, heterogeneous, anionic polysaccharide obtained as dried secretions from the aerial parts of this plant. It consists of a water-swellable component called bassorin and a water-soluble component called tragacanthin. Tragacanth has been used as a stabilizer, emulsifier, and thickener in products in the food, pharmaceutical, and cosmetic industries [156]. A previous study revealed that an aqueous solution of the gum possesses some antibacterial activity [157] and the gum is used in some hair gels and conditioners. In addition to containing a high concentration of phenolic and flavonoid derivatives [155,158], its roots contain saponins such as astragaloside I and IV, which have been reported to exhibit anticancer and anti-inflammatory effects [159,160]. Although direct mechanistic studies on *A. gossypinus* are lacking, the known biological activities of these phytochemicals are consistent with potential cosmetic effects of the extract through modulation of the Nrf2/ARE antioxidant pathway, inhibition of NF-κB-mediated inflammation, and suppression of MMPs, thereby potentially reducing UV-induced oxidative stress, inflammation, and collagen degradation associated with skin aging [159]. Given these characteristics and its history of use, this species can be considered as a promising candidate for application in the cosmetics industry.

### 3.20. A. microcephalus Willd.

This is one of the Astragalus species distributed in the Irano-Turanoian region [161]. Studies have shown that this plant contains flavonoids [162,163] including liquiritigenin, which has been reported to exhibit anti-inflammatory, antioxidant, and UV-protective properties [164], and sulfuretin, which has been reported to possess antioxidant and anti-inflammatory activities [17]. Akbari et al. [165] evaluated the wound healing effects of methanolic extracts of *A. microcephalus* root. The study was conducted using both in vitro assays with normal human dermal fibroblasts (NHDF) and an in vivo Wistar rat model. The results showed that the extracts exhibited wound healing activity with low toxicity, which the authors associated with the presence of flavonoids, especially isoflavonoids.

## 4. Future Perspectives

The exploration of the genus *Astragalus* for cosmeceutical use is at a pivotal stage. While the phytochemical potential of species beyond *A. membranaceus* is evident, several strategic research directions should be prioritized to bridge the gap between ethnobotanical knowledge and modern industrial applications. Future research should focus on establishing rigorous standardization protocols for extracts. Identifying specific “marker compounds” for each of the 20 species (such as astragaloside IV or specific flavonoids like trifolin) is essential for ensuring batch-to-batch consistency and facilitating regulatory compliance in the global cosmetic market. Furthermore, there is a critical need to advance beyond in vitro and animal models to human clinical trials. Studies should prioritize assessing the long-term topical safety and efficacy of these diverse species, particularly regarding their potential effects on skin barrier repair and chronic inflammatory conditions such as atopic dermatitis.

Many bioactive compounds in *Astragalus*, such as flavonoids and saponins, can be sensitive to environmental degradation or have limited skin penetration. Investigating advanced delivery technologies, such as liposomal encapsulation, nanoemulsions, or nanofibers, could significantly enhance the stability and bioavailability of these phytochemicals in topical formulations. While pathways such as Nrf2, NF-κB, and MAPK have been identified as relevant to the biological activities of extracts from some species, deeper mechanistic studies are required for the more understudied members of the genus. Future work should employ “omics” technologies (transcriptomics and proteomics) to map the full regulatory landscape of *Astragalus* extracts in human skin cells. Given that some species, such as *A. exscapus* subsp. *transsilvanicus*, are rare or endangered, sustainable sourcing is a priority. Future perspectives should include the use of plant tissue culture, bioreactor cultivation, or metabolic engineering to produce high-value secondary metabolites without depleting wild populations. Research into the synergistic effects between *Astragalus* polysaccharides and their phenolic counterparts could lead to multifunctional products that provide simultaneous hydration, anti-aging, and UV protection.

## 5. Conclusions

Research on the cosmeceutical potential of *Astragalus* is promising; however, its translation into evidence-based cosmetic applications is currently limited by the scarcity of clinical studies, uncertainty regarding optimal dosage and long-term topical safety, variability in extract composition and standardization, limited characterization of molecular mechanisms of action, and a lack of head-to-head comparisons with established cosmetic active ingredients. Among the approximately 3000 species of *Astragalus*, only a limited number have been reported in cosmetic applications, most notably *A. membranaceus* and *A. gossypinus*. While *A. membranaceus* has been incorporated into skincare and anti-aging formulations and *A. gossypinus* into hair-care products, the present review highlights the cosmeceutical potential of 18 additional species, including *A. sinicus*, *A. exscapus* L. subsp. *transsilvanicus*, *A. caragana*, *A. verus* Olivier, *A. gummifer*, *A. plumosus* var. *krugianus*, *A. glycyphyllos*, *A. mongholicus*, *A. monspessulanus*, *A. cruciatus*, *A. dasyanthus*, *A. melanophrurius*, *A. gombiformis*, *A. macrocephalus* subsp. *finitimus*, *A. siliquosus*, *A. gyzensis*, *A. strictispinus*, and *A. microcephalus*. These species exhibit diverse phytochemical profiles, particularly rich in flavonoids, saponins, and polysaccharides, and have demonstrated biological activities relevant to cosmetic applications, including antioxidant, anti-inflammatory, anti-tyrosinase, photoprotective, collagen-promoting, wound-healing, and hair-growth-related effects. However, the strength of evidence varies considerably among species, with particularly limited data available for *A. siliquosus* and *A. gyzensis*.

Overall, the available evidence suggests that selected *Astragalus* species may serve as promising sources of bioactive ingredients for applications such as photoprotection, moisturization, skin brightening, anti-aging, and hair care. Nevertheless, the available evidence does not yet establish the clinical efficacy or long-term safety of most species or their extracts for cosmetic use. Importantly, the absence of reported toxicity in the reviewed studies should not be interpreted as evidence of established safety, particularly in the absence of standardized toxicological and long-term topical safety assessments. Future research should therefore prioritize well-designed clinical trials, systematic evaluation of topical safety and optimal dosage, comprehensive characterization and standardization of extracts, and clarification of molecular mechanisms of action, including pathways such as Nrf2 and MAPK signaling. Comparative studies against established cosmetic active ingredients would further help define the relative efficacy and practical value of *Astragalus*-derived ingredients. Addressing these gaps will be essential for the development of reproducible, evidence-based, and regulatory-compliant *Astragalus*-derived cosmetic ingredients and for determining their potential role in sustainable cosmeceutical formulations. Table 1 summarizes the characteristics of selected *Astragalus* species as potential candidates for cosmetic applications.

## Figures and Tables

**Table 1:** Characterization of *Astragalus* species as potential candidates for use in cosmetic products.

Species	Compounds	Effects	Pathways/Evidence Status	References
*A. membranaceus*	Astragaloside IV, formononentin, calycosin	Anti-wrinkle, moisturizing, skin brightening, rejuvenating, hair and follicle booster, and anti-inflammatory	Directly reported: NF-κB/MAPK in specific experimental models; skin-cell evidence for collagen/MMP-1; constituent-level: PKA/CREB, p38 MAPK; Nrf2-related evidence	[51,53,54,58,60,61,62,63]
*A. sinicus*	Myricetin, ampelopsin	Antioxidant, collagen stimulator and TYR inhibitor, and elastase; reported improvements in skin indices	Directly reported: AKT/ERK and Wnt in human follicle dermal papilla cells; additional anti-inflammatory activity in cytokine-induced keratinocytes	[30,65,66,68]
*A. exscapus* subsp. *transsilvanicus*	Linoleic acid, palmitic acid, Astragaloside IV, Astragaloside VII	Antimicrobial, anti-aging, anti-inflammatory, moisturizing, anti-melanoma	No species-specific intracellular pathway experimentally established	[70,71,72]
*A. caraganae*	Rutin, chlorogenic acid, isoquercitrin	Anticancer, anti-inflammatory, anti-allergy, anti-blemish, and photoprotective	Constituent-level/proposed: NF-κB and MAPK-related signaling; not validated for the whole extract	[74,75]
*A. verus*	Thymol, palmitic acid, phytol	Antifungal, antibacterial, anti-inflammatory, photoprotective, anti-aging, hair grower, and wound-healing	Constituent-level: NF-κB/MAPK for thymol in other models; no species-specific pathway established	[77,80,81,82,83,84,85,86]
*A. gummifer*	Astragaloside IV	Anti-inflammatory, skin regeneration, softener, and moisturizer	No species-specific NF-κB/Nrf2/VEGF mechanism established	[87,90,91]
*A. plumosus* var. *krugianus*	Askrugianoside A, oleifolioside B	Anti-aging and antioxidant	No species-specific intracellular pathway established	[92,93,94]
*A. glycyphyllos*	Quercetin, rutin, stearic acid, palmitic acid, linoleic acid	Anti-aging, anti-inflammatory, antifungal, and moisturizer	Constituent-level/proposed: NF-κB/MAPK and Nrf2; not established for the whole extract	[9,39,40,95,96,97,98]
*A. mongholicus*	Astragaloside IV, rosmarinic acid, zelaic acid, formonentin, coumaric acid	Anti-aging, anti-acne, anti-inflammatory, and collagen stimulator	Reported in specific models: TGF-β/Smad and Nrf2/ARE; pathway evidence depends on preparation/model	[60,100,101,103]
*A. monspessulanus*	Trifolin, rutin, cosmosiin	Anti-inflammatory, photoprotective, anti-TYR, and antifungal	No single species-specific intracellular pathway established; constituent-level evidence available	[105,106,107,108,110,111]
*A. cruciatus*	Nicotiflorin, daucosterol, narcissi	Anti-inflammatory and antioxidant	Proposed/constituent-level: Nrf2 and NF-κB; direct whole-extract pathway validation is lacking	[112,113,114,115,116,117,118]
*A. dasyanthus*	Tartaric acid, arabinose, quercetin, kaempferol, bassorin, citric acid, malic acid, glycyrrhizin	Anti-aging, anti-inflammatory, and pH regulator	Constituent-level evidence only; no species-specific pathway established	[119,120,121,122,123,124,125,126,127]
*A. melanophrurius*	Ferulic acid, rosmarinic acid, p-coumaric acid, chlorogenic acid, verbascoside	TYR inhibition, anti-TYR, anti-aging, antibacterial, anti-inflammatory, anticancer, anti-wrinkle, and wound-healing	Constituent-level/proposed: Nrf2/HO-1, NF-κB, TRPV3, and MITF; whole-species validation remains limited	[129,130,131,132,133,134,135]
*A. gombiformis*	Cirsiliol, quercitrin, ferulic acid, silymarin	Anti-inflammatory, antibacterial, and anticancer	Constituent-level/proposed: NF-κB/MAPK for cirsiliol; AHR-Nrf2 for silymarin in related models	[136,139]
*A. macrocephalus* subsp. *finitimus*	Hyperoside, *p*-coumaric acid, apigenin	Anti-wrinkle, antibacterial, anti-inflammatory, anticancer, and anti-TYR	Constituent-level/proposed: Nrf2/HO-1, NF-κB, and MITF; not uniformly demonstrated in the whole extract	[140,141,142,143,144]
*A. siliquosus*		Anti-TYR and antioxidant	Direct evidence: enzyme-level TYR inhibition; no species-specific intracellular pathway established	[145,147]
*A. gyzensis*	Uronic acid	Moisturizer	No species-specific pathway established	[148]
*A. strictispinus*	Salicylic acid, cycloasalgenin, rutin, cycloastragenol, apigenin, hesperidin	Anti-aging, anti-inflammatory, anti-acne, anti-blemish, and photoprotective	Constituent-level/proposed mechanisms; whole-species pathway validation is limited	[12,70,149,150,151,152,153,154]
*A. gossypinus*	Salicylic, caffeic, cinnamic, coumaric, ferulic, gallic acids, rutin, catechine, quercetin, kaempferol, daidzein, naringenin, apigenin, myricetin, epigallocatechin gallate, epicatechin, astragaloside I and IV	Antioxidant, anticancer, anti-inflammatory, anti-mutagenic, anti-aging, antibacterial, anti-acne, emulsifier, photoprotective	Proposed from constituent literature: Nrf2/ARE and NF-κB; direct extract-level mechanistic evidence is limited	[155,159]
*A. microcephalus*	Liquiritigenin, sulfuretin, calycosin, 7-hydroxyflavone	Wound-healing, anti-inflammatory, stimulator of collagen and fibroblast production	No species-specific intracellular pathway established	[17,162,165]

## Data Availability

The data presented in this study are available on request from the corresponding author.
